# Aberrant *ARID5B* expression and its association with Ikaros dysfunction in acute lymphoblastic leukemia

**DOI:** 10.1038/s41389-018-0095-x

**Published:** 2018-11-12

**Authors:** Zheng Ge, Qi Han, Yan Gu, Qinyu Ge, Jinlong Ma, Justin Sloane, Guofeng Gao, Kimberly J. Payne, Laszlo Szekely, Chunhua Song, Sinisa Dovat

**Affiliations:** 1 Department of Hematology, Zhongda Hospital Southeast University, Institute of Hematology Southeast University, 210009 Nanjing, China; 2 International Cooperative Leukemia Group and International Cooperative Laboratory of Hematology, Zhongda Hospital Southeast University, 210009 Nanjing, China; 3 State Key Laboratory of Bioelectronics, School of Biological Science and Medical Engineering, Southeast University, 210096 Nanjing, China; 4 Abington Hospital, Jefferson Health, Abington, PA 19001 USA; 5 Department of Pediatrics, Pennsylvania State University Medical College, Hershey, PA17033 USA; 6 Department of Pathology and Laboratory Medicine, University of California-Davis Medical Center, Sacramento, CA 95817 USA; 7 Department of Pathology and Human Anatomy, Loma Linda University, Loma Linda, CA 92350 USA; 8 Department of Medicine, Laboratory of Clinical Pathology and cytology, Karolinska University Hospital, Solna, L2:04, SE-171 76 Stockholm, Sweden

## Abstract

Mutations and single nucleotide polymorphisms of AT-rich interactive domain-containing protein 5B (ARID5B) are involved in the oncogenesis of acute lymphoblastic leukemia (ALL) and treatment outcomes. However, ARID5B expression and clinical significance in ALL remain unclear. We found *ARID5B* is significantly down-regulated in ALL compared to healthy bone marrow controls. ARID5B also interacts with PHD finger protein 2 (PHF2). Low expression of *ARID5B* (*ARID5B*^*low*^) or *ARID5B* and *PHF2* (*ARID5B*^*low*^*PHF2*^*low*^) is correlated with the markers of cell proliferation and poor prognosis in ALL patients. Ikaros directly regulates ARID5B expression in ALL. Restoring Ikaros function by Casein Kinase II inhibition also promotes ARID5B expression through recruitment of trimethylation of lysine 4 on histone H3 (H3K4me3) at its promoter region. In summary, our data show that aberrant expression of ARID5B and PHF2 is related to leukemic cell proliferation and several poor prognostic markers. Our data indicate ARID5B^low^ expression, particularly ARID5B^low^PHF2^low^ expression, is linked to Ikaros dysfunction and involved in the oncogenic effect of high-risk ALL, which may represent a high-risk subgroup of ALL.

## Introduction

The complex of AT-rich interactive domain-containing protein 5B (ARID5B) formed with PHD finger protein 2 (PHF2) induces the demethylation of lysine 9 di-methylation on histone H3 (H3K9me2) to activate the transcription of the target genes^1,2^. ARID5B is widely expressed throughout the human body. However ARID5B dysfunction appears to be closely linked with leukemia^2–10^. *ARID5B* mutations /SNPs (single nucleotide polymorphisms) are reported to be involved in the oncogenesis of acute lymphoblastic leukemia (ALL) and treatment outcome^3–10^. Reports also showed that ARID5B knockdown impairs cell cycling by up-regulating p21, and contributes to methotrexate (MTX) and 6-mercaptopurine (6-MP) resistance and eventual relapse^3–10^. We observed that PHF2 expression is down-regulated in ALL cells. Until now, the clinical significance of *ARID5B* expression has not been determined in ALL patients.

Ikaros, the product of the IKZF1 gene, is not only an essential transcription factor for lymphocyte development but also a key suppressor in leukemogenesis^11,12^. The profile of Ikaros’ global genomic binding has been identified in ALL cells^13–16^. Ikaros binding sites are observed at the *ARID5B* promoter using ChIP-seq. We reported that Casein Kinase II (CK2) inhibition could restore the leukemia suppressor activity of Ikaros and CK2 inhibitors are the activator of the Ikaros function^12–15^. We demonstrated that once activated, Ikaros regulates the expression of gene targets by histone modification mechanism, and that it can induce transcription activation of its target genes by recruitment of H3K4me3 in ALL^13–17^. Here, we studied *ARID5B* expression in patients with ALL and discovered that *ARID5B*^*low*^ expression is linked to the markers of leukemia cell proliferation and that ARID5B^low^PHF2^low^ expression is possibly a poor prognostic indicator in patients with ALL. We also show that *ARID5B*^*low*^ expression is closely related with IKZF1 gene deletion in B-ALL. Our data manifest that Ikaros directly modulates *ARID5B* expression and that restoring Ikaros function in ALL cells from patients promotes *ARID5B* expression through the acquisition of H3K4me3. Our results identify the oncogenic effects of the *ARID5B*^*low*^*PHF2*^*low*^ expression pattern and its association with Ikaros dysfunction, which may reveal a novel high-risk subgroup of ALL.

## Results

### Laboratory characteristics in patients with low *ARID5B* expression

**Fig. 1:**
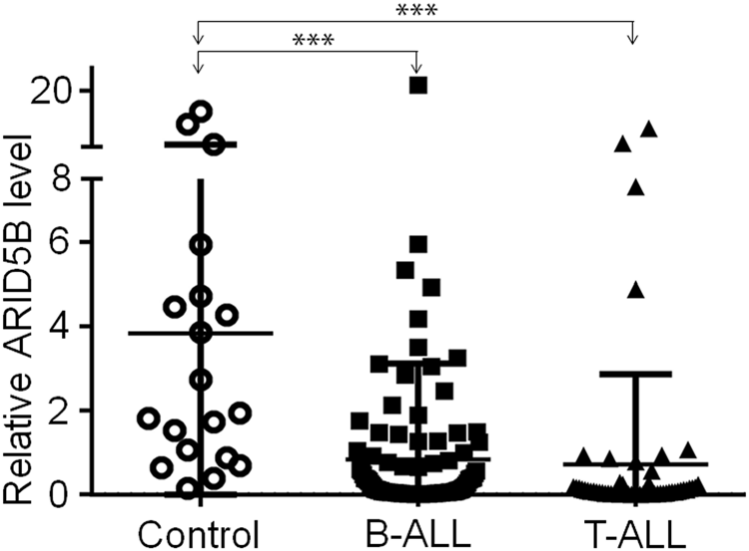
*ARID5B* expression in ALL. *ARID5B* expression in B-ALL (*N* = 123) and T-ALL (*n* = 57) and normal bone marrow controls (*n* = 19). ***p < 0.001

The mRNA level of *ARID5B* in the adult ALL patients’ bone marrow samples was significantly lower than those in normals (Fig. 1). Similarly, the cohort studies in B-cell ALL (B-ALL) and T-cell ALL (T-ALL) (Fig. S1) showed that *ARID5B* expression in mRNA levels was significantly lower than that in B cells from healthy controls (Fig. S1). The laboratory features were compared in patients with B-ALL by dividing them into two groups: high *ARID5B* mRNA levels (ARID5B^high^) or low *ARID5B* mRNA levels (*ARID5B*^low^) (Table 1 and Table S1). A significantly higher median percentage of BM blasts (90.0% vs. 84.6%, *P* = 0.037) and a significantly higher percentage of cases positive for CD34 (CD34+), the stem cell marker (88.8% vs. 37.5%, *P* = 0.000) or CD33 (CD33+), the myeloid marker (48.5% vs. 25.0%, *P* = 0.046) were observed in patients with low *ARID5B* mRNA level compared to that of high level. Similarly, low *ARID5B* mRNA level in patients was correlated with a higher frequency of cases positive for expression of Ikaros isoform 6 (*IK*6+), the gene product of the most common *IKZF1* deletion isoform (42.5% vs. 20.0%, *P* = 0.042), and also a lower median hemoglobin (HGB) and platelet (PLT) count compared to patients with high *ARID5B* expression (Table S1). We discovered that B-ALL patients with low *ARID5B* expression represented a cohort with a significantly higher percentage of those requiring more than 4 weeks to reach complete remission (CR), a poor prognostic indicator in ALL, (51.4% vs. 16.0%, *P* = 0.002), as compared to that with high *ARID5B* expression (Table S1). However, among T-ALL patients, the low and high *ARID5B* expression groups did not show significantly different representation in the patient cohort (data not shown).

**Table 1:** Significant correlation of *ARID5B*^*l*ow^*PHF2*^*l*ow^ expression with high-risk markers in B-ALL

Characteristics	*ARID5B* ^*l*ow^ *PHF2* ^*l*ow^	*non-ARID5B* ^*l*ow^ *PHF2* ^*l*ow^	Univariate analyses (Chi-Square Tests)	Multivariate analyses (Multivariate Cox model)	
			*P* value	*P* value	HR(95% CI)
*IKZF1* deletion (IK6 expressing) (%)	49.3	15.8	0.001	0.001	0.062 (0.013–0.298)
Blasts (%) median (range) bone marrow	91.2 (59.0–100.0)	82.4 (28.0–98.0)	0.000	0.038	0.005 (0.000–0.742)
Extramedullary infiltration (%) spleen	50.0	22.9	0.008	0.964	1.032 (0.264–4.029)
Stem cell marker CD34 + (%)	88.2	55.6	0.000	0.135	0.370 (0.100–1.362)
Myeloid marker CD33 + (%)	50.9	28.6	0.036	0.711	1.307 (0.317–5.381)
Time to CR after treatment is > 4 weeks (%)	53.0	21.2	0.003	0.002	0.132 (0.036–0.478)

### Correlation of *ARID5B*^*low*^*PHF2*^*low*^ expression with clinical features in B-ALL

ARID5B and PHF2 interact with one another^1,2^. We found that ARID5B mRNA levels were positively correlated with *PHF2* expression in the microarray analysis of B-ALL and T-All cohort studies (Fig. S2). We analyzed the co-occurrence of low-level *ARID5B* and low-level *PHF2* expression (*ARID5B*^*low*^*PHF2*^*low*^) and its association with clinical features (Table S2). *ARID5B*^*low*^*PHF2*^*low*^ expression was correlated to a higher percentage of cases with splenomegaly (50.0% vs. 22.9%, *P* = 0.008) and a lower PLT count (10^9^/L) (32.0 vs. 58.5, *P* = 0.020) when compared to patients that were non-*ARID5B*^*low*^*PHF2*^*low*^ (Table S2). Moreover, the percentage of bone marrow blasts, a direct marker of high leukemic cell proliferation, showed significantly higher in *ARID5B*^*low*^*PHF2*^*low*^ than that in none-*ARID5B*^*low*^*PHF2*^*low*^(91.2% vs. 82.4%, *P* = 0.000), and multivariate analyses confirmed this result (HR 0.005, 95% CI [0.000, 0.742]; *P* = 0.038) (Table 1).

We observed the correlation between *ARID5B*^*low*^*PHF2*^*lo*w^ expression and several poor prognostic markers. A higher percentage of the *ARID5B*^*low*^*PHF2*^*low*^ cases were positive for CD34 (88.2% vs. 55.6%, *P* = 0.000) or CD33 (50.9% vs. 28.6%, *P* = 0.036). Importantly, the low expression cohort also showed a significantly higher frequency of Ik6 + cases (49.3% vs. 15.8%, *P* = 0.001), and a substantially higher percentage of patients with a CR time ≥ 4 weeks when compared to the none-*ARID5B*^*low*^*PHF2*^*low*^ expression cohort and confirmed by multivariable analysis (Table 1).

We looked over the relationship between *ARID5B* expression and survival. No significant differences were identified in the overall survival (OS) of the patients with *ARID5B*^*low*^ or *ARID5B*^*low*^*PHF2*^*lo*w^ expression as compared to those in the *ARID5B*^*high*^ or none-*ARID5B*^*low*^*PHF2*^*low*^ cohorts, respectively (Fig. S3 and Fig. S4). However, we did observe a trend towards a shortened relapse-free survival (RFS) in patients with *ARID5B*^*low*^ expression, especially the *ARID5B*^*low*^*PHF2*^*low*^ cohort, compared to those with *ARID5B*^*high*^ or non-*ARID5B*^*low*^*PHF2*^*low*^ expression, respectively (Fig. S3 and Fig. S4).

### *The ARID5B* expression is regulated by Ikaros in ALL

**Fig. 2:**
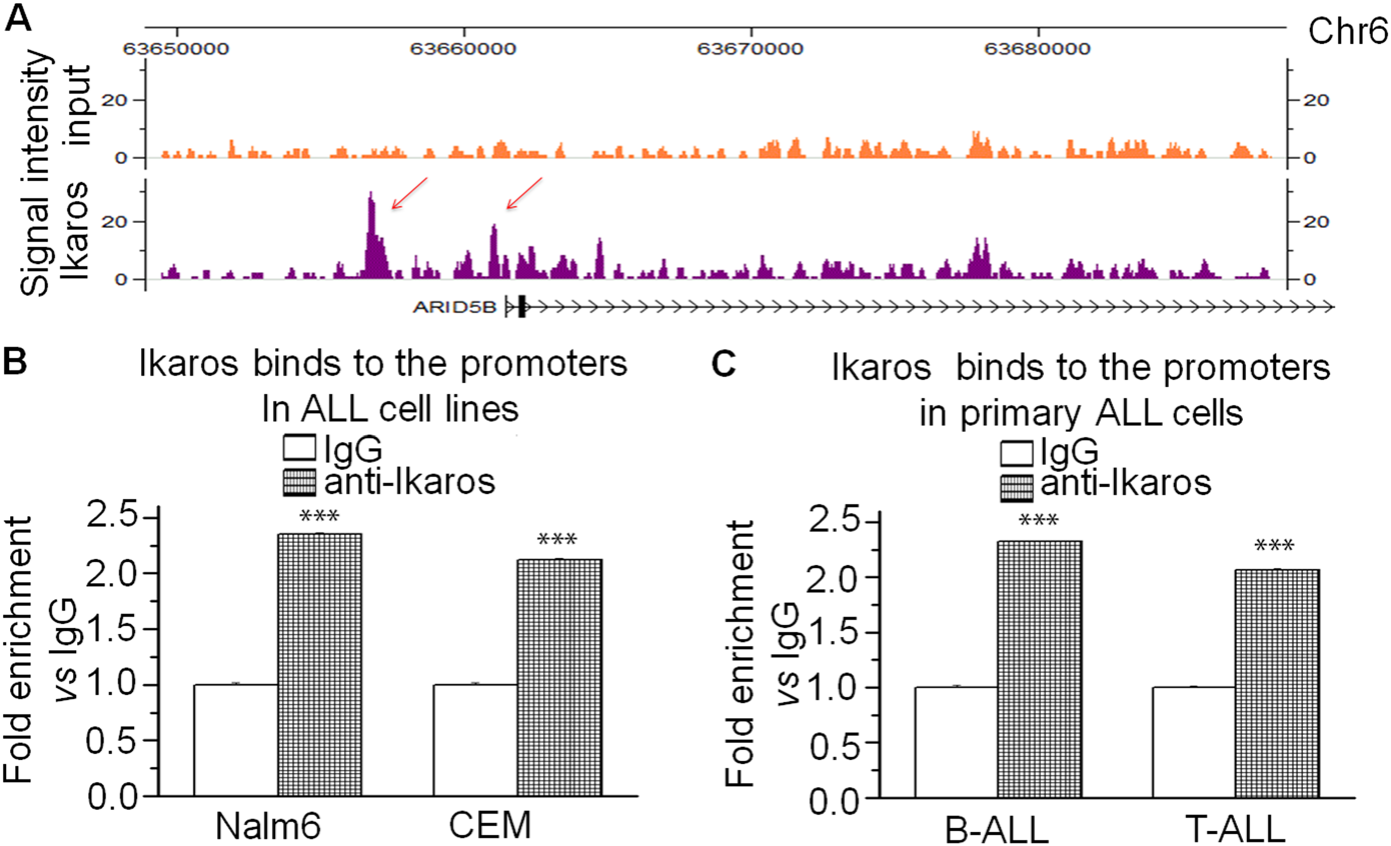
Ikaros binding sites at *ARID5B* promoter in B-ALL cells were determined by ChIP-seq (**a**). **b, c** Ikaros binding at *ARID5B* promoter was validated by qChIP assay in **b** ALL cell lines and (**c**) primary ALL cells. Graphed data are the mean ± SD of triplicates representative of one of 3 independent experiments (**a, b**) or 3 patient samples (**c**). ***p < 0.001

**Fig. 3:**
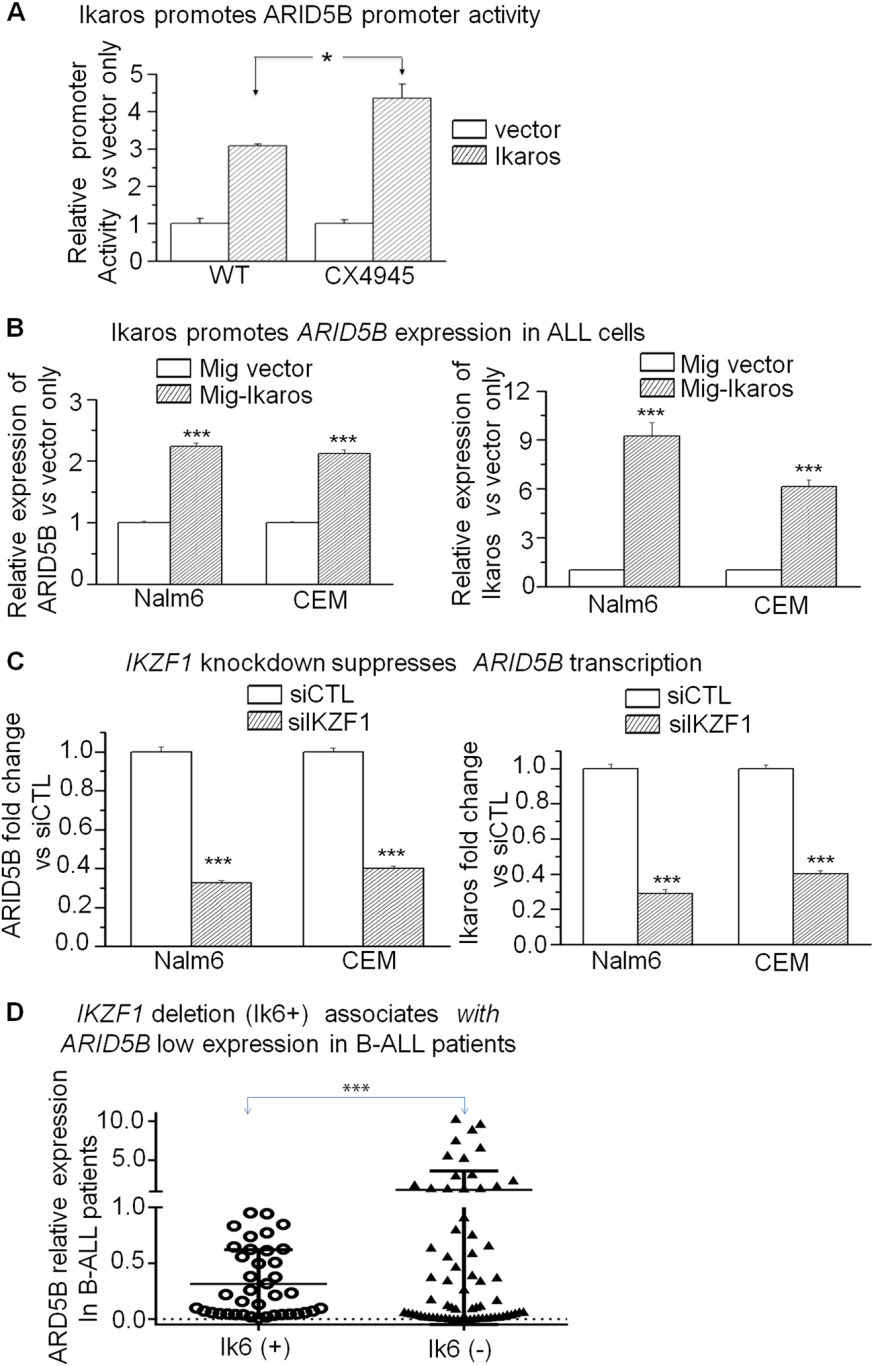
Ikaros induces *ARID5B* expression in ALL. **a** The activity of the *ARID5B* promoter was assessed with transfection of *Ikaros* or control vector in HEK293 cells with or without the CK2 inhibitor, CX-4945, by luciferase reporter assay; (**b**) Nalm6 and CEM cells were transduced to express Ikaros (Mig-Ikaros) or with empty vector (Mig vector) and assessed by qPCR for expression of *ARID5B*. Graphed indicates the relative *ARID5B* expression; (**c**) Nalm6 and CEM cells were treated with *IKZF1* siRNA (si-*IKZF1)* or control siRNA (siCTL) and assessed by qPCR for expression of *ARID5B*. Graphed is the relative expression of *ARID5B*; (**d**) Patients that were positive (n = 39) vs. negative (*n* = 68) for Ik6, the expressed gene product of the *IKZF1* deletion, were assessed by qPCR for expression of *ARID5B*. The *ARID5B* expression in **a**–**c** expresses as the mean ± SD of triplicates representative of one of 3 independent experiments. *p < 0.05, ***p < 0.01

To understand the underlying mechanism of *ARID5B* low expression in ALL, we studied Ikaros binding sites present in the *ARID5B* promoter region by ChIP-seq assay, in Nalm6 (Fig. 2a) and primary B-ALL cells (Fig S5)^13,14^. qChIP assay confirmed Ikaros recruitment at *ARID5B* promoter in the leukemia cell lines (Fig. 2b) and primary cells (Fig. 2c). These results suggest Ikaros has a direct regulation on *ARID5B* transcription. We further showed that Ikaros increases promoter activity of *ARID5B* using the luciferase reporter assay (Fig. 3a). Ikaros transduction of Nalm6 and CEM cells results in the significant increase of *ARID5B* expression (Fig. 3b). Conversely, efficient Ikaros knockdown significantly decreased *ARID5B* mRNA level in both of these cell lines (Fig. 3c).

### Association of *IKZF1* deletion with *ARID5B* low expression in B-ALL patients

Microarray analysis in B-ALL and T-ALL cohorts^18–20^ showed the positive correlation of *IKZF1* mRNA levels with *ARID5B* expression (Fig. S6). A significant *ARID5B* low expression was observed in B-ALL patients that were *IK*6 + (0.3153 ± 0.0938 vs. 1.2052 ± 0.58441, *P* = 0.02439) (Fig. 3d), which is consistent with our finding that the *ARID5B*^*low*^ cohort has a significantly higher percentage of *IK*6 + cases in B-ALL (Table S1). These data reveal the contribution of the *IKZF1* genetic defects to low *ARID5B* expression in B-ALL patients.

### CK2 inhibitor CX-4945 promotes *ARID5B* transcription by enhancing Ikaros activity

**Fig. 4:**
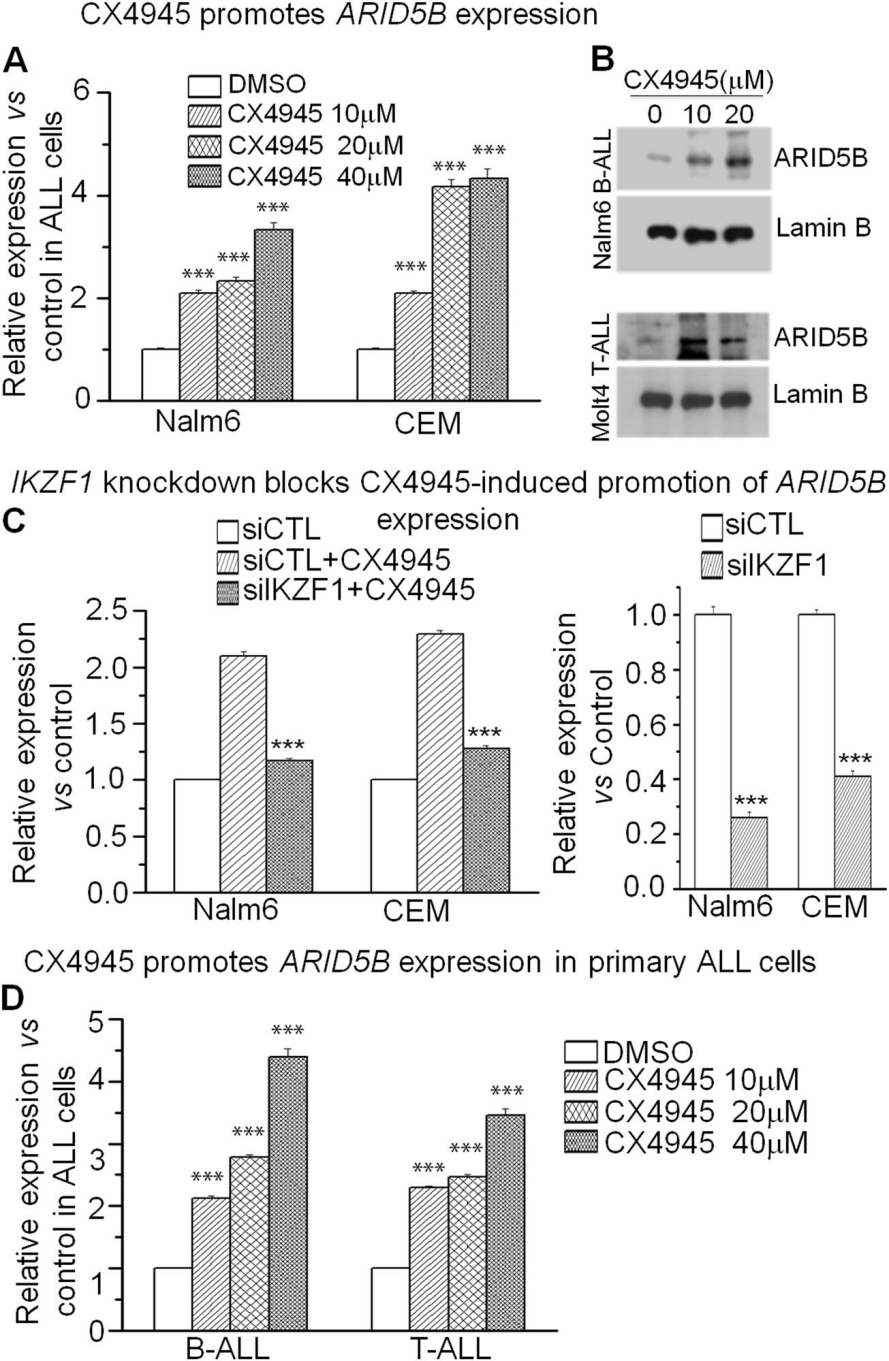
Ikaros dependence on CX-4945 promoting *ARID5B* expression. **a** Treatment with CX-4945 induces an increase in *ARID5B* expression in Nalm6 and CEM cells; ***p < 0.001 compared to DMSO control. **b** Protein levels of ARID5B as evaluated by Western blot in the indicated cells that were incubated with different doses (10 μM, 20 μM) of CX-4945 or DMSO control (0) for 48 h. Lamin B was used for loading control. **c** Effect of Ikaros shRNA knockdown on the CX-4945-induced promotion of *ARID5B* expression. ***p < 0.01 compared to siCTL + CX4945 group; (**d**) CX-4945 promotes *ARID5B* expression in primary ALL cells; ***p < 0.001 compared to the control. Graphed data in A-D represents the mean+/– SD of triplicates representative of one of 3 independent experiments

Our previous studies show that the CK2 inhibitor, CX-4945, can restore Ikaros’ tumor suppressor activity^13^. CX-4945 treatment further improves Ikaros-mediated increase of *ARID5B* promoter activity when compared to that without treatment (Fig. 3a). Using qPCR, we showed that CX-4945 treatment in Nalm6 and CEM cells enhances *ARID5B* mRNA level in a dose-dependent manner (Fig. 4a). Western blot data showed that CX-4945 treatment also increases the ARID5B protein level as compared to that of DMSO control in the two cell lines (Fig. 4b). Moreover, Ikaros knockdown significantly attenuates CX-4945-induced increases in the ARID5B mRNA level in ALL cell lines (Fig. 4c). The effect of CX-4945 on *ARID5B* mRNA levels is also observed in primary B-/T-ALL cells (Fig. 4d). These results indicate that CX-4945 promotes *ARID5B* transcription by increasing Ikaros function as tumor suppressor in ALL.

### Increasing Ikaros activity by CK2-inhibition promotes H3K4me3 occupancy at the *ARID5B* promoter

**Fig. 5:**
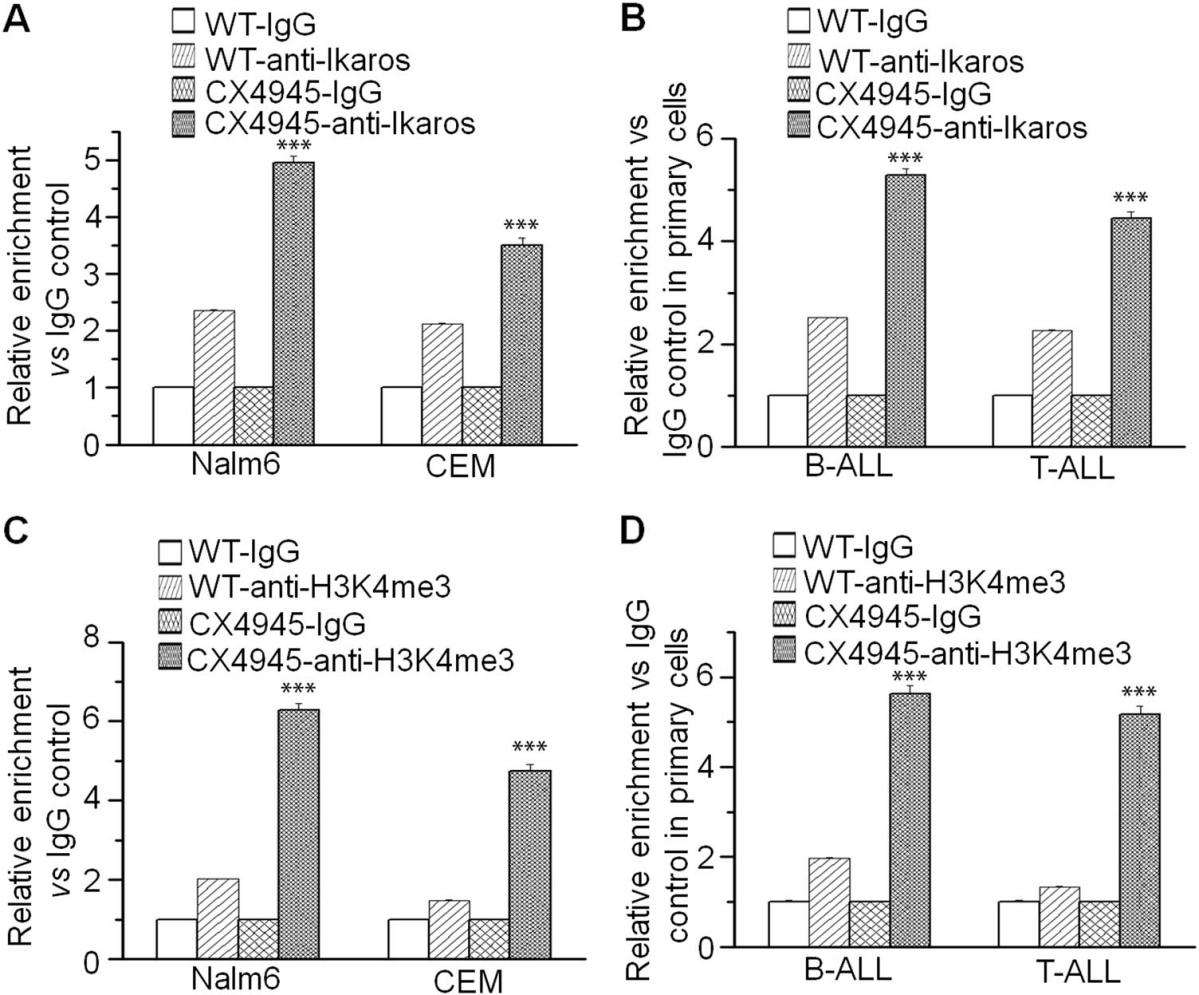
Chromatin switches upon CX-4945 treatment. Indicated cell lines and primary cells were treated with 10 μM CX-4945 or with DMSO control and evaluated by qChIP for Ikaros binding (**a**, **b**) and the H3K4me3 histone mark (**c**, **d**) at the *ARID5B* promoter in the indicated cells. ***p < 0.001 compared to WT-anti-Ikaros control. Graphed data in **a**–**d** are the mean+/– SD of triplicates representative of one of 3 independent experiments or 3 patient samples

Ikaros regulates target gene expression through histone modification mechanism^14^. To explored if Ikaros regulates *ARID5B* expression also via epigenetic mechanisms, we performed ChIP assays to amplify the resulting *ARID5B* promoter sequences. Our data show that the Ikaros binding to the *ARID5B* promoter is significantly increased upon CX-4945 treatment not only in Nalm6 and CEM cells (Fig. 5a), but also in primary B-/T-ALL cells (Fig. 5b). CX-4945 treatment also results in the increases of H3K4me3 recruitment at the *ARID5B* promoter in the cell lines (Fig. 5c), and in the primary cells (Fig. 5d).

## Discussion

The *ARID5B* gene product is widely expressed in the human tissue and has been linked to leukemia^2–10,21–25^. *ARID5B* mutations /SNPs are linked to the ALL development and adverse treatment outcomes^4^. Aberrant *ARID5B* expression halts B-lymphocyte maturation in the developing fetus and contributes to leukemogenesis^21^. However, the mRNA level of *ARID5B* in primary ALL and its association with clinical findings have not been reported. Our findings show the correlation of *ARID5B* expression with a difference in clinical features in ALL. We previously showed that *PHF2* is down-regulated in ALL^26^. We saw that *ARID5B* and *PHF2* expression were positively correlated in ALL and that *ARID5B*^*low*^*PHF2*^*low*^ expression is associated with leukemic cell proliferation (high bone marrow blasts and splenomegaly, low HGB and PLT), as well as a poor prognosis (high percentage of Ik6+, ≥4 weeks to reach CR upon treatment, and CD33+) in B-ALL patients. Next, we showed that Ik6 expression, the most common *IKZF1* deletion is significantly linked to *ARID5B* low expression in B-ALL. We further demonstrated that *ARID5B* is a direct gene target of Ikaros, the *IKZF1* gene product, in ALL. Finally, our study identifies a potential high-risk subgroup of ALL with *ARID5B*^*low*^*PHF2*^*low*^ expression and reveals the oncogenic effect of *ARID5B*^*low*^*PHF2*^*low*^ expression and its correlation with Ikaros dysfunction in ALL.

There have been many reports that SNPs affect gene expression. In addition to reports that *ARID5B* SNPs increase the risk of ALL, several reports also indicate that both *ARID5B* and *IKZF1* SNPs are positively associated with ALL^4–9,22–25,27–29^. However, no reports are involved in exploring the relationship between *ARID5B* SNPs and *ARID5B* expression. Our data reveal that the *IKZF1* genetic defect (Ik6 expression) is associated with *ARID5B* low expression and that Ikaros directly promotes *ARID5B* expression. This information also suggests that the association of *ARID5B* and *IKZF1* SNPs with an increased risk of ALL may result from the low expression of *ARID5B* and *IKZF1*, although the effects of *ARID5B* and *IKZF1* SNPs on their expression need to be further investigated.

Transcriptional and epigenetic abnormalities are key factors in oncogenesis. The ARID5B-PHF2 complex is involved in the activation of tumor suppressors, such as p53, through its effect on methylation^30^. Our data shows that the correlation between *ARID5B*^*low*^*PHF2*^*low*^ expression and leukemic cell proliferation, with poor prognostic markers in B-ALL. We also found that restoring Ikaros function by CK2 inhibition could increase *ARID5B* and *PHF2* expression, as well as increase H3K4me3 binding at the promoter region. This data is the first to indicate the regulatory mechanism underlying *ARID5B* gene expression. It also suggests that targeting transcriptional and epigenetic abnormalities is a potential strategy for developing effective new therapeutics for ALL.

In conclusion, we show that *ARID5B*^*low*^*PHF2*^*low*^ expression is correlated with markers for leukemic cell proliferation and poor outcome. Our results further reveal the effects of *ARID5B*^*low*^*PHF2*^*low*^ expression on ALL oncogenesis and identify a possible subgroup of high-risk ALL with characterization of both *ARID5B*^*low*^*PHF2*^*low*^ expression and Ikaros dysfunction.

## Materials and methods

### Patient samples and therapies

The 164 bone marrow samples were obtained from patients with ALL, diagnosed at our institutes between 2008 and 2016. All of the patients (107 B-ALL and 57 T-ALL), ages 12–77 years old, were recruited in the cohort study, with diagnoses based on the 2008 revision of the WHO Diagnosis and Classification of ALL. As controls, 19 normal bone marrow samples were used. Following the Declaration of Helsinki, the informed consent was documented by all patients before recruitment.

As previously published (CALLG2008)^31^, patients received either VDCLP therapy, which consists of Vincristine (V), Daunorubicin (D), Cyclophosphamide (C), L- Asparaginase (L), and Prednisone (P), or CAT therapy, which contains C, Cytarabine (A), Thioguanine (T), high-dose Mitoxantrone (M), and methotrexate/L-Asparaginase (Met/Asp) for induction or early induction. For late consolidation, VDLP or the combined therapy of CVCED (E: Epipodophyllotoxin and D: Dexamethasone), and high-dose Met/Asp, E and A were utilized. Lastly, 6-Mercaptopurine and M were used during maintenance therapy. Imatinib was also added to regimens for patients with Ph (+) ALL starting on day 15 of induction therapy.

The Ethics Committee of Zhongda Hospital Southeast University and the First Affiliated Hospital of Nanjing Medical University, Nanjing, China approved this study.

### Cytogenetic and molecular analyses

Ikaros 6 (*IK*6), the most common expression product from the *IKZF1* deletion, was detected as previously described^32^. Briefly, the isolated genomic DNA with QIAamp DNA Blood Mini Kit (Qiagen, Germantown, MD, USA) was utilized for performing the genomic PCR amplification for detection of *IKZF1* deletion on exons 4–7 (△4–7). The flanking deletion breakpoints of *IK*6 was characterized by direct sequencing of the resulted PCR products. Cytogenetics was also analyzed as described^32^.

### Quantitative Real-time PCR (qPCR) assay

For qPCR of patient samples, the real-time PCR system (StepOne Plus 7500) from Applied Biosystem-Thermofisher (Foster, CA, USA) was utilized. Briefly, cDNA was generated from total RNA (1.0 μg) using SuperScript II first-Strand synthesis kit (Invitrogen, Carlsbad, CA, USA) with poly d(T)20 primers. The genes’ mRNA level was analyzed from the resulting cDNAs on the machine by using the specific primer of each gene. Primers for the qPCR of ARID5B are: Sense: 5′- TCTTAAAGGCAGACCACGCAA −3′, Anti-sense: 5′- TGCCATCGGAATTGTTGTTGG −3′. Primers for qPCR of 18 s rRNA were as previously reported^13–15,17,31^. Two groups of the cohorts were divided as patients with high or those with low *ARID5B* expression (4th quartile vs. 1st–3rd quartiles), and SPSS 20.0 was utilized for determination of the cut-off value. ARID5B or PHF2 expression was calculated in the individual sample by a formula as previously described^15–17,31–33^. The formula was determined from the value of a scatter Ct graph in a serially diluted template standard. *ARID5B* or *PHF2* expression level was normalized to housekeeping gene 18 s rRNA with a formula of *ARID5B*/*18* *s* rRNA or *PHF2*/*18* *s* rRNA.

The qPCR assay was also used to analyze *ARID5B* mRNA levels in the cell lines. Results of drug treatment, Ikaros overexpression, or *IKZF1* knockdown were divided by those acquired with housekeeping gene18s rRNA and expressed as fold change over DMSO or vector controls.

### Cell culture

The previously described Nalm6 cell line^34^, is verified by the American Type Culture Collection (ATCC, Manassas, VA). The CCRF-CEM (CEM) and HEK 293 T cell lines were obtained from ATCC. DMEM (Cellgro, Tewksbury, MA, USA), supplemented with 10% FBS and 1% L-glutamine (Cellgro, Tewksbury, MA, USA) was used for culture of HEK 293 T cells; and the 10% FBS (Hyclone, Logon, Utah, USA) supplemented RPMI 1640 medium (Cellgro, Tewksbury, MA, USA) for culturing Nalm6, CEM, and primary human B-/T-ALL cells at 37 °C in a 5% CO2 humidified atmosphere. CX-4945 was obtained from Selleckchem (S2248, Houston, USA). Cells with or without CX-4945 treatment were used for total RNA isolation, as well as western blot.

### Plasmid construction and retroviral gene transduction

Human full-length Ikaros (IKZF1) cDNA was cloned into the retroviral vector, MSCV-IRES-GFP (MIG) with BglII and EcoRI site^15,34,35^. The plasmids were transiently transfected into amphotropic packaging HEK 293 cell lines and the retroviruses were generated and concentrated as described^15,34,35^. Cells plated on a 24-well plate at 4 × 10E5 cells/well were centrifuged 1400×g in retroviral supernatants plus 12.5 mg/ml polybrene, at 32 °C, for 1 h. The cells were further cultured in fresh media at 37 °C, 5% CO2 incubator for 3 days. The GFP(+) cells were sorted with BD FACS Aria SORP high-performance sorter (BD Biosciences, Sparks, MD, USA), and the sorted cells are cultured for further RNA isolation and ChIP assay.

### Luciferase assay

LightSwitch luciferase reporter constructs for promoters of *ARID5B* were purchased from Active Motif-SwitchGear Genomics (Carlsbad, CA, USA). The transfection-ready promoter plasmid, or pLightSwitch-Rom vector, was transfected with Ikaros in pCDNA3.1 vector or vector only into HEK293 cells and the transient luciferase assay was done with or without 10 μM CX-4945 according to Switchgear Genomics manual by a luminometer as previously described^14–17,31–36^. Briefly, *ARID5B* promoter-reporter plasmids and pcDNA3.1-Ikaros or pcDNA3.1 vector were delivered into HEK293 cells in 24-well plates in a 1:3 ratio with the transfection reagent, lipofectamine 2000 (Invitrogen, Carlsbad, CA, USA). The cells were lysed 24 h after transfection in 100 μl of lysis buffer (Active Motif-SwitchGear Genomics, Carlsbad, CA, USA). Half of the lysate was used for luciferase activity measurement on a GloMax Luminometer (Promega, Madison, WI, USA). The luciferase activity was determined as fold change of the values from the cells transfected with promoter construct relative to ones obtained from pLightSwitch-Rom vector-only control cells. Ikaros effect on the promoter activity was presented as a ratio of Ikaros-induced luciferase activity over that of the vector. The graphed data was the average of triplicates which is one representative of 3 independent experiments.

### Western blot assay

Nuclear extracts were isolated by osmotic swelling and homogenization from the cells treated with different doses of CX-4945 and DMSO as controls^1,3,14,15,25^. Protein concentrations were determined by the quantitative Bradford assay. Total protein (20 μg) of each sample was used for the western blot assay as previously described^13,15^. ARID5B protein expression was detected with the anti-ARID5B antibody (ab226776, Abcam, Cambridge, MA, USA) and Lamin B was detected by the anti-Lamin B1 antibody (VPA00119, Bio-Rad, USA) as a loading control.

### Quantitative chromatin immune precipitation (qChIP)

Chromatin from cells treated with CX-4945 was incubated with antibodies against Ikaros^14,15,25^. Cells were cross-linked in the 1% formaldehyde solution on ice and the cross-link reaction was ceased with 0.125 M glycine. The chromatin for Ikaros ChIP assay was prepared from 2 × 10E7 Nalm6 or CEM cells or primary leukemia cells (4–10 × 10E6) and fragmented with a Bioruptor (Diagenode, Denville, NJ) to obtain the average DNA size of 400 bp as previously described^14,15,25^. For ChIP assays, the chromatin was incubated with Dyneabeads-coated affinity-purified rabbit polyclonal anti-Ikaros antibody^14,15,25^ or normal rabbit IgG (Abcam, ab46540) as the control. The protein/DNA complexes were isolated with a Magnetic separator (Invitrogen, Carlsbad, CA, USA) and extensively washed with RIPA buffer. The ChIP’d DNA was eluted and reversely crosslinked. The resulted samples were further treated with proteinase K digestion, phenol/chloroform extraction, and RNaseA incubation. A QIAquick PCR Purification kit (QIAGEN) was used for recovering the ChIP’d DNAs. Enrichment of Ikaros-bound-ARID5B promoter in the ChIP’d DNA sample vs. that with normal rabbit IgG (ab171870, Abcam, Cambridge, MA, USA) as a control was measured by qPCR with the primers at ARID5B promoter(forward: 5′- GCAGTCGCTGTCCGTTCAA −3′, reverse: 5′- CAAGTGAGCAGTGCACACACA −3′)^14,15,25^. At least three technical replicates were performed for each assay. The relative Ikaros binding at the ARID5B promoter is expressed as the fold change of Ikaros-bound DNA vs. that of rabbit IgG controls. H3K4me3 qChIP assay was done using the same protocol as Ikaros qChIP, with the anti-H3K4me3 antibody (ab8580, Abcam, Cambridge, MA, USA), except using 1 × 10E7 cells for them as we previously reported^14,15,25^.

### *IKZF1* shRNA knockdown

A set of 4 pGFP-V-RS constructs containing unique human Ikaros (ikzf1) 29mer shRNA were purchased from Origene (Rockville, MD, USA). The optimal gene knockdown shRNA plasmid from the 4 constructs was tested and selected using the Neon Transfection System (Invitrogen, Carlsbad, CA, USA) for further studies. After transfection for one day, cells were observed with 80–90% (green cells) transfection efficiency and more than 95% cell viability. The cells incubated with 10 μM CX-4945 or non-treatment DMSO control for 2 days were harvested for total RNA isolation. The cells transfected with a scrambled shRNA (29-mer) vector were used as a control. Ikaros level was evaluated in the cells by qPCR with IKZF1 specific primer as previously reported^15,35^.

### Statistical analyses

Median differences between the groups in the cohort study were tested utilizing a Mann–Whitney *U*-test. The univariate and multivariate Cox models were used for statistical analysis of frequency differences. The Kaplan-Meier analysis with the log-rank test was utilized to judge the significance for RFS and OS. The date of diagnosis was the initial point for OS, and RFS was started at the time of declared remission to that of patients achieving complete remission (CR). Living patients were counted on for survival at follow up. Data were graphed as mean value ± SEM (standard error of the mean). Analysis of variance (ANOVA) or Student t-test was used to evaluate the statistical significance for comparisons of two groups or comparing multiple groups, respectively.

## Electronic supplementary material

Supplemental Tables

Supplemental Figures

## Data Availability

In accordance with local health research ethics protocols, the patient datasets for the current study are not publicly accessible; however, it may be available from the corresponding author.
