# Supplementary material for: Aberrant ARID5B expression and its association with Ikaros dysfunction in acute lymphoblastic leukemia
Source: Oncogenesis. 2018 Nov 12;7(11):84. doi: 10.1038/s41389-018-0095-x (PMC6232140; doi:10.1038/s41389-018-0095-x)
Supplement: Supplementary file 1 — Supplemental Tables [file 41389_2018_95_MOESM1_ESM.docx]

**Table S1**  Association of *ARID5B* expression with clinical features in B-ALL

| **Characteristics** | ***ARID5B*^low^**  **(N=81)** | ***ARID5B*^high^**  **(N=26)** | ***P* value** |
| --- | --- | --- | --- |
| Age（years） |  |  |  |
| median（range） | 39.0(12.0-77.0) | 23.5(12.0-70.0) | 0.162 |
| Sex（%） |  |  |  |
| male | 56.8 | 53.8 | 0.792 |
| WBC，×10^9^/L |  |  |  |
| median（range） | 31.6(0.9-487.9) | 36.1(0.7-299.9) | 0.899 |
| Hemoglobin(HGB),g/L |  |  |  |
| median（range） | 85.0(45.0-150.0) | 127.0(44.0-157.0) | 0.010 |
| PLT,×109/L |  |  |  |
| median（range） | 33.5(2.0-292.0) | 77.0(8.0-292.0) | 0.024 |
| LDH（u/L） |  |  |  |
| median（range） | 667.0(125.0-8702.0) | 953.5(222.0-7142.0) | 0.273 |
| Blasts（%）median（range） |  |  |  |
| bone marrow | 90.0（28.0-100.0） | 84.6（38.0-98.0） | 0.037 |
| peripheral blood | 70.0（0.0-98.0） | 65.5（0.0-96.0） | 0.244 |
| Stem cell marker CD34+ (%) | 88.8 | 37.5 | 0.000 |
| Myeloid marker,% |  |  |  |
| CD13+ | 49.3 | 39.1 | 0.401 |
| CD33+ | 48.5 | 25.0 | 0.046 |
| Extramedullary infiltration（%） |  |  |  |
| liver | 7.5 | 8.7 | 1.000 |
| spleen | 45.7 | 22.7 | 0.052 |
| lymph node | 40.7 | 30.4 | 0.370 |
| Genetics（%） |  |  |  |
| *IKZF1* deletion (IK6) | 42.5 | 20.0 | 0.042 |
| BCR/ABL1 fusion gene(Ph+) | 48.7 | 33.3 | 0.185 |
| complex karyotype | 17.0 | 16.7 | 1.000 |
| Time to reach CR after treatment>4 weeks(%) | 51.4 | 16.0 | 0.002 |

**Table S2**  Association of *ARID5B* and *PHF2* expression with clinical features in B-ALL

| **Characteristics** | **ARID5B^low^ PHF2^low^**  **(N=68)** | ***non-ARID5B^l^*^ow^*PHF2^low^***  **(N=39)** | ***P* value** |
| --- | --- | --- | --- |
| Age（years） |  |  |  |
| median（range） | 39.0（12.0-77.0） | 27.0（12.0-70.0） | 0.464 |
| Sex（%） |  |  |  |
| male | 54.4 | 59.0 | 0.647 |
| WBC，×10^9^/L |  |  |  |
| median（range） | 31.0（0.9-487.9） | 37.2（0.7-380.3） | 0.787 |
| HGB,g/L |  |  |  |
| median（range） | 87.0（45.0-150.0） | 99.0（44.0-157.0） | 0.126 |
| PLT,×109/L |  |  |  |
| median（range） | 32.0（2.0-292.0） | 58.5（4.0-292.0） | 0.020 |
| LDH（u/L） |  |  |  |
| median（range） | 590.0（125.0-8702.0） | 932.0（181.0-7313.0） | 0.165 |
| Blasts（%）median（range） |  |  |  |
| bone marrow | 91.2（59.0-100.0） | 82.4（28.0-98.0） | 0.000 |
| peripheral blood | 76.0（0.0-98.0） | 67.0（0.0-96.0） | 0.071 |
| Stem cell marker CD34+ (%) | 88.2 | 55.6 | 0.000 |
| Myeloid marker,% |  |  |  |
| CD13+ | 51.8 | 38.2 | 0.212 |
| CD33+ | 50.9 | 28.6 | 0.036 |
| Extramedullary infiltration（%） |  |  |  |
| liver | 7.5 | 8.3 | 1.000 |
| spleen | 50.0 | 22.9 | 0.008 |
| lymph node | 39.7 | 36.1 | 0.720 |
| Genetics（%） |  |  |  |
| *IKZF1* deletion (IK6) | 49.3 | 15.8 | 0.001 |
| BCR/ABL1 fusion gene(Ph+) | 46.2 | 43.2 | 0.776 |
| complex karyotype | 17.4 | 16.0 | 1.000 |
| Time to reach CR after treatment>4 weeks(%) | 53.0 | 21.2 | 0.003 |
