# Supplementary material for: Aberrant ARID5B expression and its association with Ikaros dysfunction in acute lymphoblastic leukemia
Source: Oncogenesis. 2018 Nov 12;7(11):84. doi: 10.1038/s41389-018-0095-x (PMC6232140; doi:10.1038/s41389-018-0095-x)
Supplement: Supplementary file 2 — Supplemental Figures [file 41389_2018_95_MOESM2_ESM.pdf]

## Supplemental Figures

**Aberrant *ARID5B* expression and its association with Ikaros dysfunction in acute lymphoblastic leukemia**

Zheng Ge[1,2,$], Qi Han[1,2], Yan Gu[1,2], Qinyu Ge[3], Jinlong Ma[1,2], Justin Sloane[4,5], Guofeng Gao[6], Kimberly J. Payne[2,7], Laszlo Szekely[2,8], Chunhua Song[2,5,$], Sinisa Dovat[2,5,$]

[1]Department of Hematology, Zhongda Hospital Southeast University, Institute of Hematology Southeast University, Nanjing 210009, China;
[2]International Cooperative Leukemia Group and International Cooperative Laboratory of Hematology, Zhongda Hospital Southeast University, Nanjing 210009, China;
[3]State Key Laboratory of Bioelectronics, School of Biological Science and Medical Engineering, Southeast University, Nanjing 210096, China;
[4]Abington Hospital, Jefferson Health, Abington, PA 19001, USA;
[5]Department of Pediatrics, Pennsylvania State University Medical College, Hershey, PA17033, USA;
[6]Department of Pathology and Laboratory Medicine, University of California-Davis
Medical Center, Sacramento, CA 95817, USA
[7]Loma Linda University, Department of Pathology and Human Anatomy, Loma Linda, CA, 92350, USA;
[8]Department of Medicine, Laboratory of Clinical Pathology and cytology, Karolinska University Hospital, Solna, L2:04, SE-171 76 Stockholm, Sweden

**Keywords:** *ARID5B*; Ikaros; *PHF*2; leukemia; epigenetics, prognosis

**Running title**: *ARID5B* low expression in ALL

[$]Co-correspondence to:
Zheng Ge, M.D., Ph.D.
Zhongda Hospital Southeast University
Department of Hematology
Nanjing 210009, China
Telephone: 86-25-83262468
FAX: 86-25-83262471
E-mail: Janege879@hotmail.com

Chunhua Song, M.D., Ph.D.
Pennsylvania State University College of Medicine
Department of Pediatrics
500 University Drive
Hershey, PA 17033, USA
Telephone: 717-531-1841
FAX: 717-531-4789
E-mail:csong@pennstatehealth.psu.edu

Sinisa Dovat, M.D., Ph.D.
Pennsylvania State University College of Medicine,
Department of Pediatrics
500 University Drive
Hershey, PA 17033, USA
Telephone: 717-531-6012
FAX: 717-531-4789
E-mail: sdovat@pennstatehealth.psu.edu

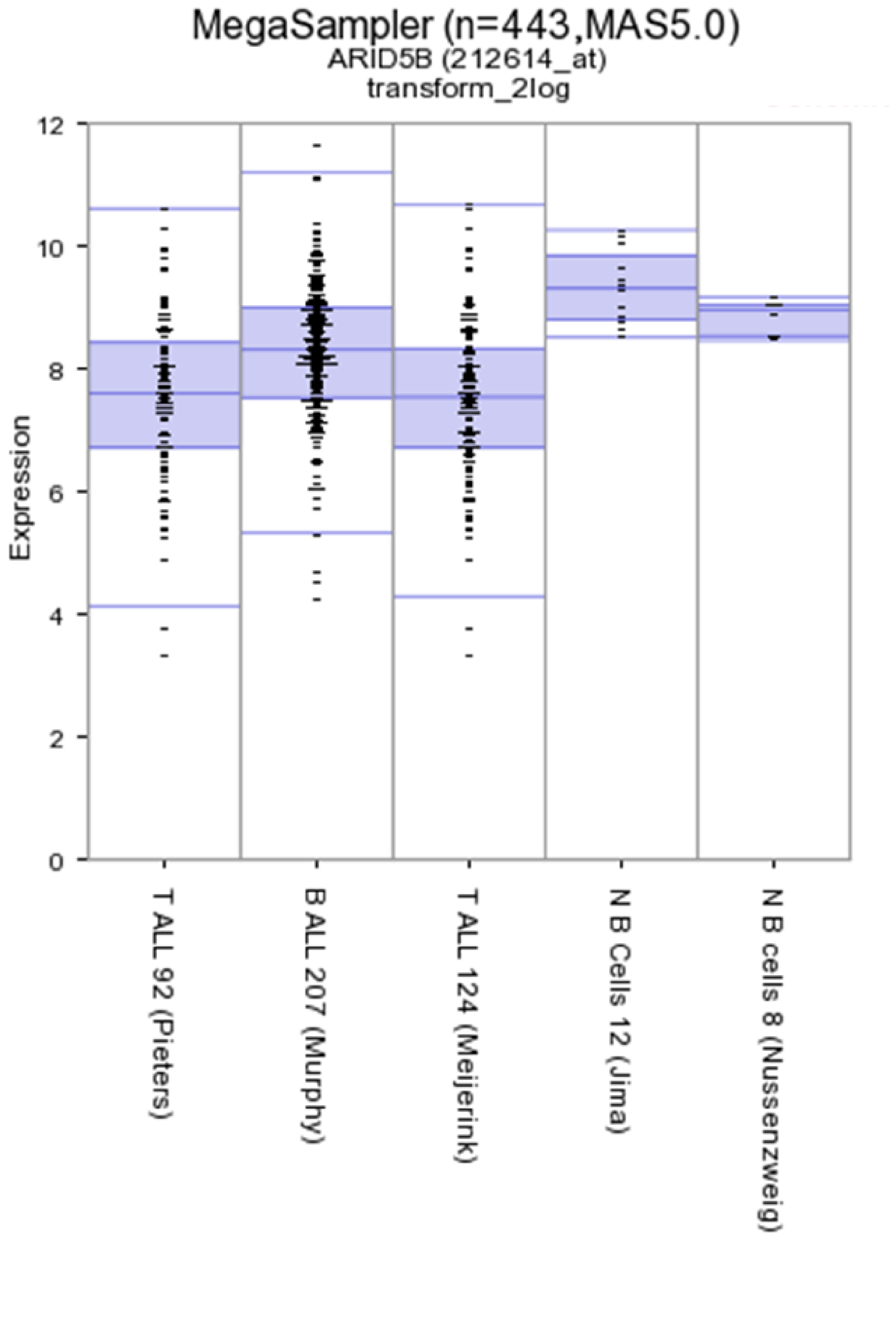


**Fig. S1** *ARID5B* expression in the reported ALL microarray cohort studies. Leukemia types are as indicated as well as normal (N) B cells. Data generated from human oncogenomics server (https://hgserver1.amc.nl/cgi-bin/r2/main.cgi) with GEO database (GSE7440, GSE11877, GSE10609, GSE26713 and GSE28491). The default one way Analysis of Variance (ANOVA) in the server was used for statistical analysis.

A

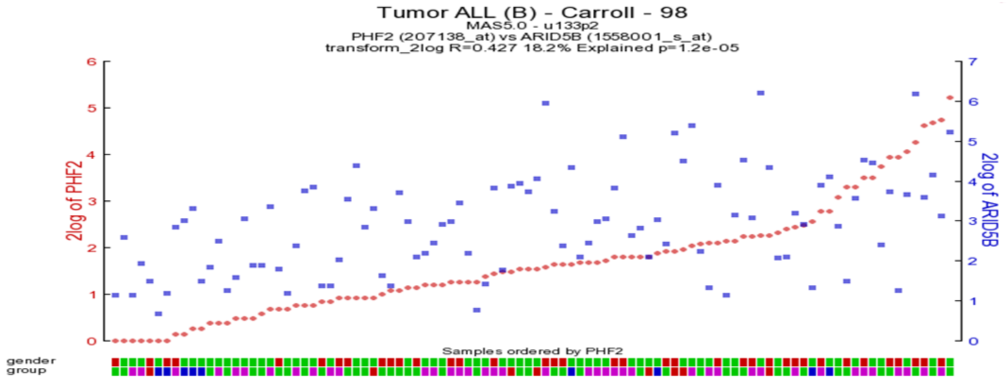


B

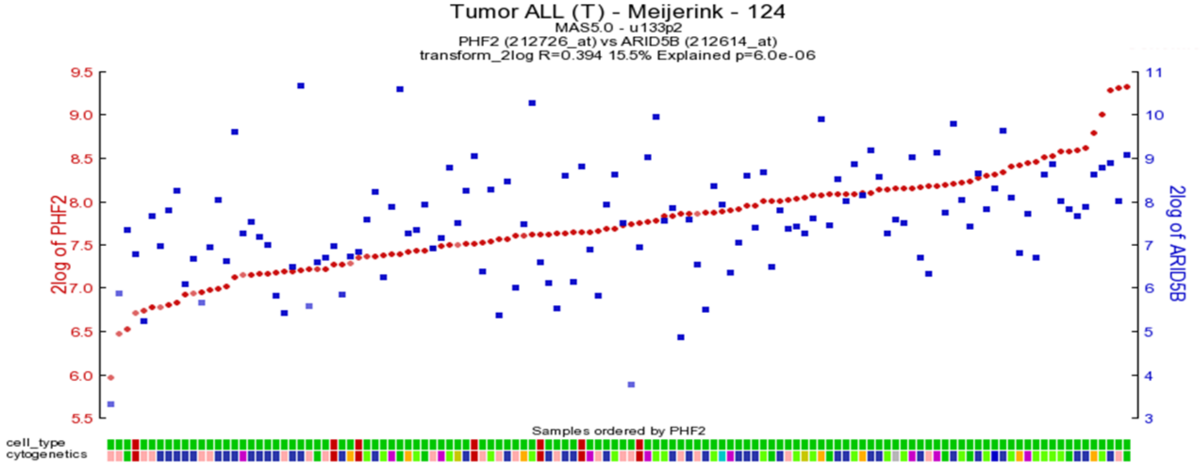


**Fig. S2 Correlation of *ARID5B* expression with *PHF2* in the cohort of (A) B-ALL and (B) T-ALL patients**. Data generated from human oncogenomics server (https://hgserver1.amc.nl/cgi-bin/r2/main.cgi) with GEO database (GSE11877 and GSE26713). The default Pearson correlation in the server was used to calculate the statistics.

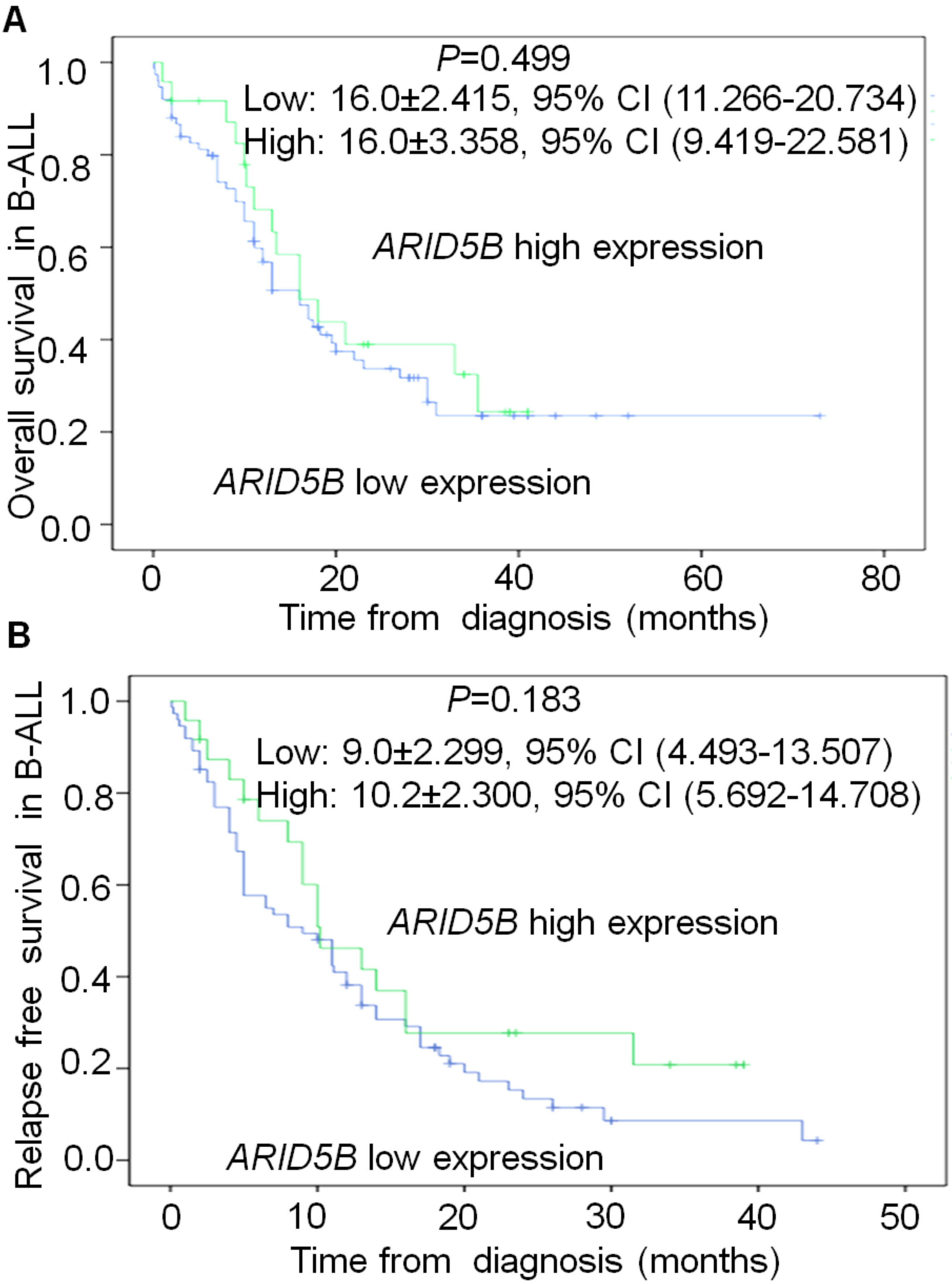


**Fig. S3** Association of *ARID5B* low expression with **(A)** overall survival and **(B)** relapse free survival in B-ALL

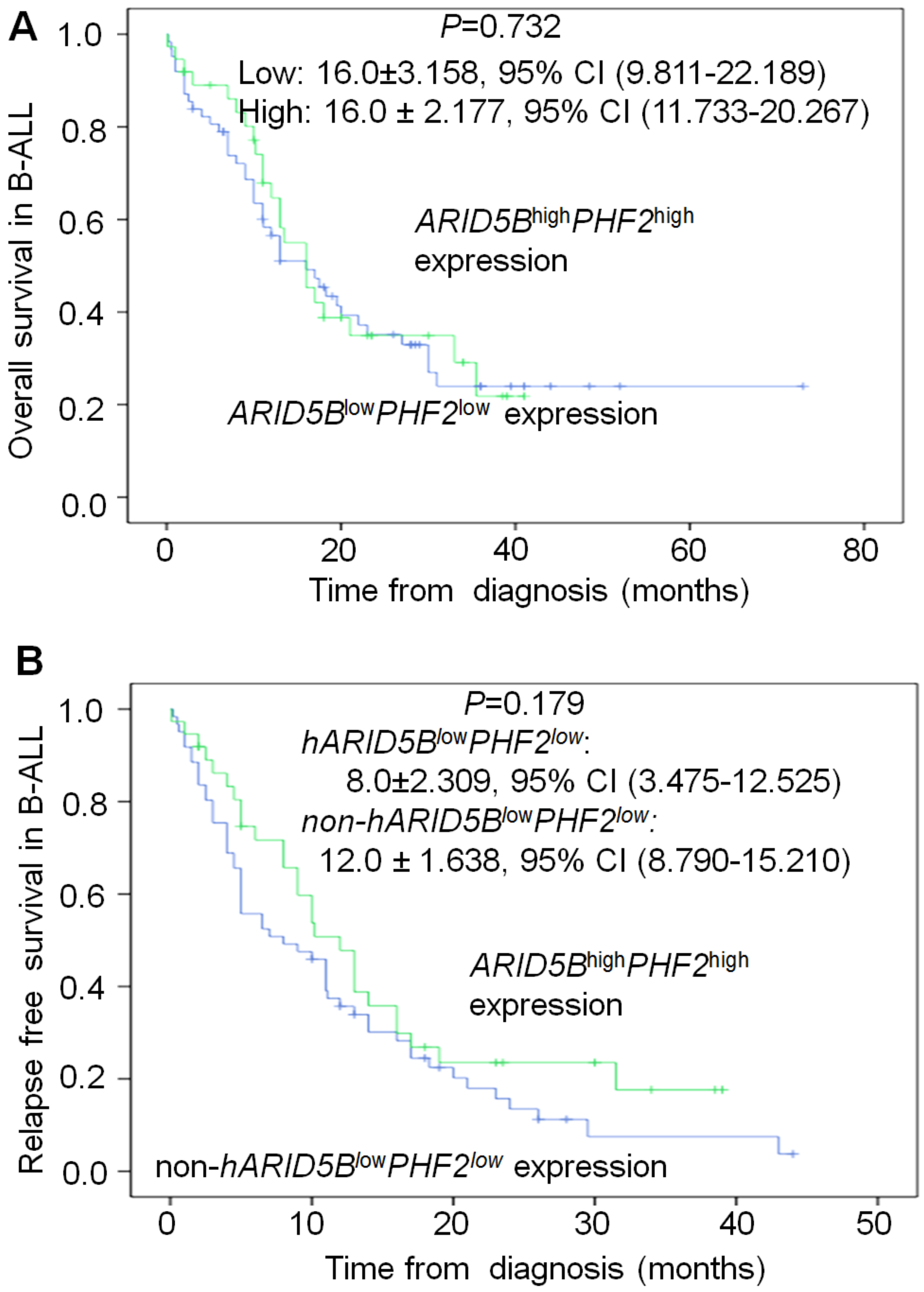


**Fig. S4** Association of *ARID5B*$^{low}$*PHF2*$^{low}$ expression with **(A)** overall survival and **(B)** relapse free survival in B-ALL

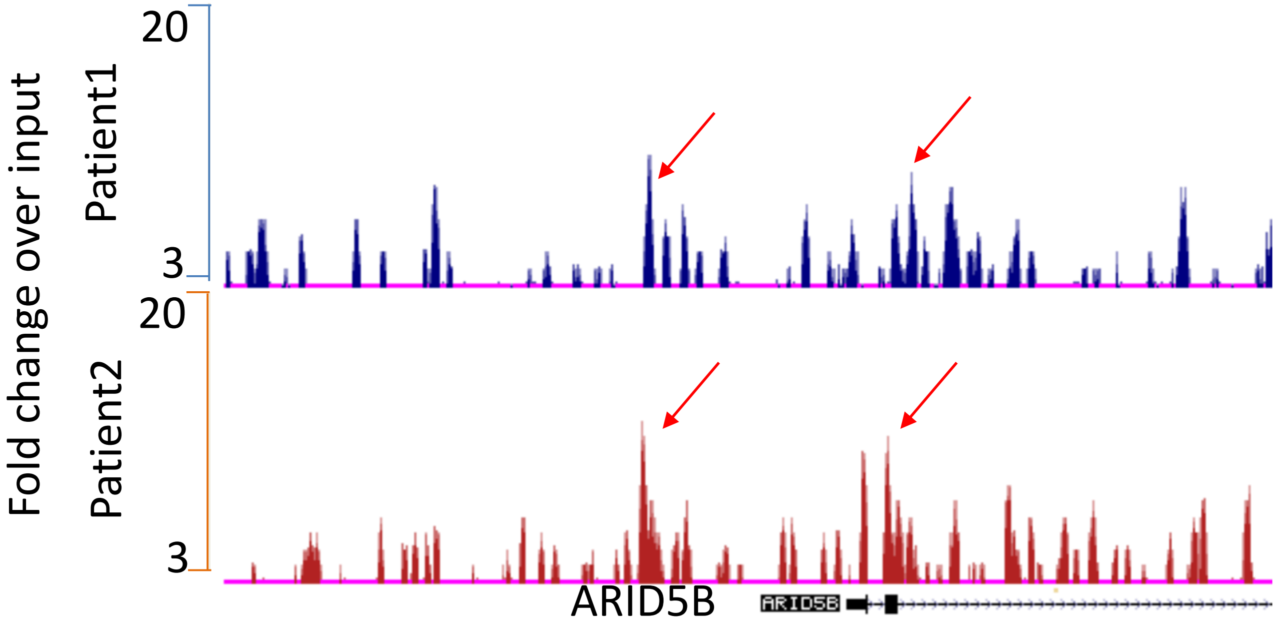


**Fig S5** Ikaros binds to the promoter of ARID5B in primary B-ALL from different patients identified by ChIP-seq.

A

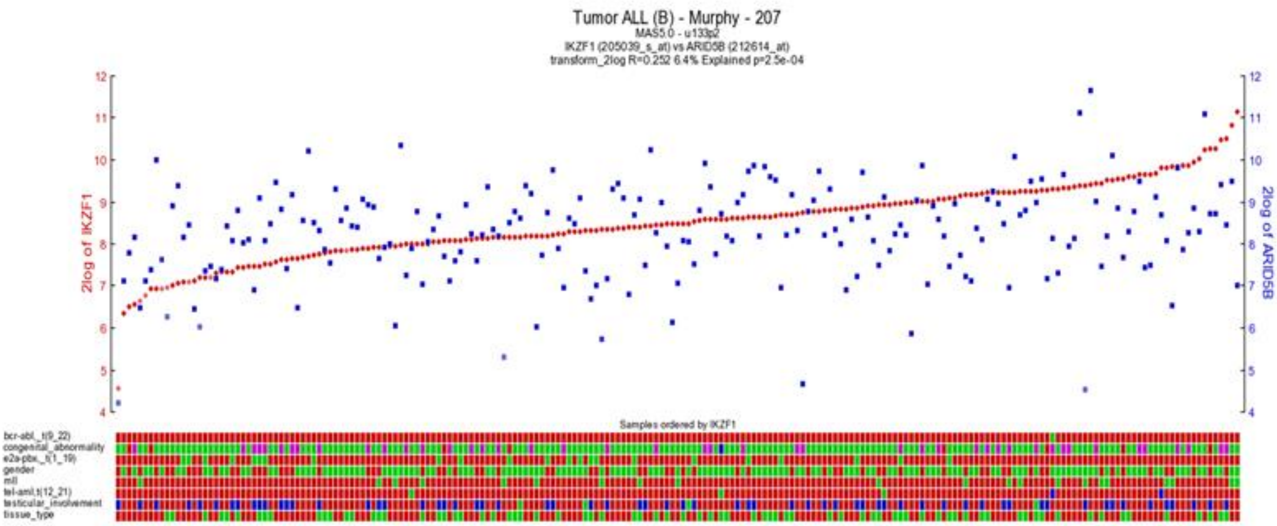


B

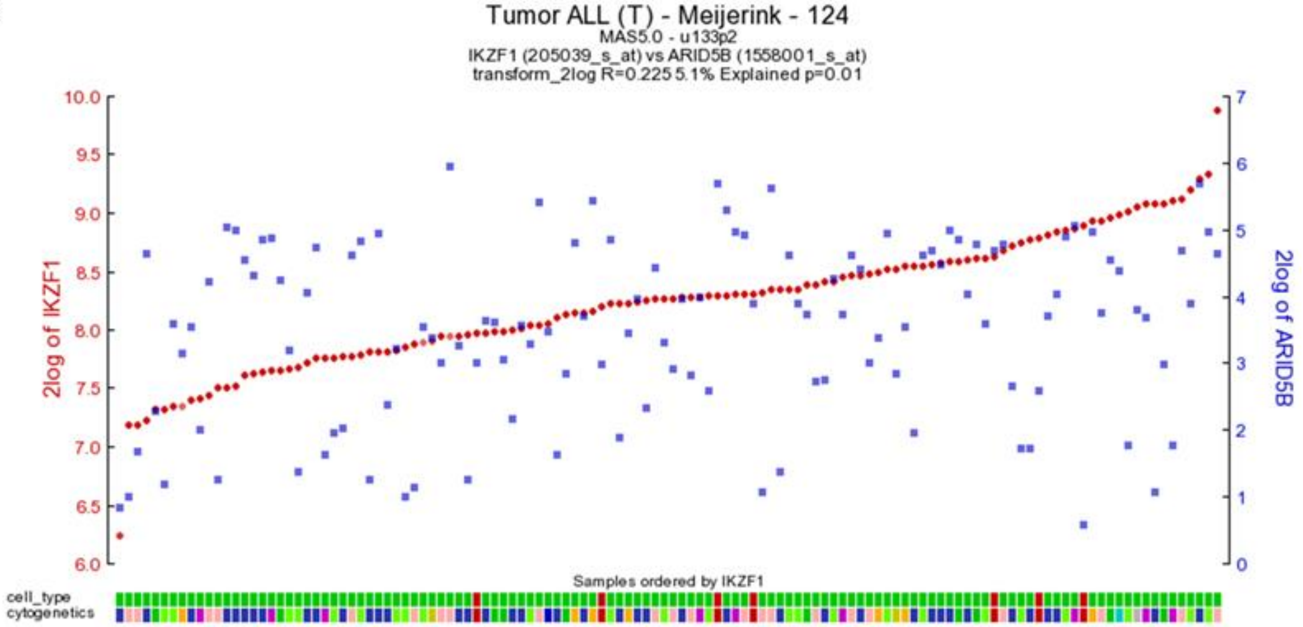


**Fig. S6 Correlation of *ARID5B* expression with *Ikaros* in the cohort of (A) B-ALL and (B) T-ALL patients.** Data generated from human oncogenomics server with GEO database (GSE11877 and GSE26713). Pearson correlation is used to calculate the statistics.
